# Typhoid Fever

**Published:** 1883-01

**Authors:** 


### TYPHOID FEVER AND MALARIAL WAVES AND THEIR RELATION.

In a recent monthly report, the Secretary of the State Board of Health of Connecticut gives statistics showing an increase in typhoid fever, and comments upon its relation to malaria as follows:

"This return of typhoid fever to prominence, and its steady increase in frequency for the last three years, is apparently a part of an extensive and comprehensive movement. As the epidemic of malaria was ushered in by a decrease, and in places almost, if not quite, a total disappearance of typhoid, this return of typhoid fever to its former importance and relative frequency is an intimation of the decrease and disappearance of malaria. The tendency toward typhoid fever commenced several years ago, and has steadily grown stronger each year, as shown by the increased prevalence, tendency to unusual frequency and severity, and the increase each year of deaths from this cause. As the decrease in the frequency of typhoid preceded the malarial wave, so its increase precedes the entire disappearance of malaria, or at least gives us some ground for hope that such a disappearance will take place. This disappearance of epidemics of malarial fever on a large scale has often been followed by an unusual prevalence of typhod fever or an extensive epidemic. The epidemics of malarial fever of 1807 and 1824, which are stated to have extended over all Europe, were followed by tyhoid fever." The writer thinks that the spread of malarial fevers over Connecticut, Massachusetts, and Rhode Island has ceased.

